# Exploring the Interactive Effects of Thymol and Thymoquinone: Moving towards an Enhanced Performance, Gross Margin, Immunity and *Aeromonas sobria* Resistance of Nile Tilapia (*Oreochromis niloticus*)

**DOI:** 10.3390/ani12213034

**Published:** 2022-11-04

**Authors:** Doaa Ibrahim, Sara E. Shahin, Leena S. Alqahtani, Zeinab Hassan, Fayez Althobaiti, Sarah Albogami, Mohamed Mohamed Soliman, Rania M. S. El-Malt, Helal F. Al-Harthi, Nada Alqadri, Mohamed Tharwat Elabbasy, Marwa I. Abd El-Hamid

**Affiliations:** 1 Department of Nutrition and Clinical Nutrition, Faculty of Veterinary Medicine, Zagazig University, Zagazig 44511, Egypt; 2 Department of Animal Wealth Development, Veterinary Economics and Farm Management, Faculty of Veterinary Medicine, Zagazig University, Zagazig 44511, Egypt; 3 Department of Biochemistry, College of Science, University of Jeddah, Jeddah 80203, Saudi Arabia; 4 Fish Disease Department, Faculty of Veterinary Medicine, Aswan University, Aswan 81528, Egypt; 5 Department of Biotechnology, College of Science, Taif University, Taif 21944, Saudi Arabia; 6 Clinical Laboratory Sciences Department, Turabah University College, Taif University, Taif 21995, Saudi Arabia; 7 Department of Bacteriology, Zagazig Branch, Animal Health Research Institute, Agriculture Research Center, Zagazig 44516, Egypt; 8 Department of Biology, Turabah University College, Taif University, Taif 21995, Saudi Arabia; 9 College of Public Health and Molecular Diagnostics and Personalized Therapeutics Center (CMDPT), Ha’il University, Ha’il 2440, Saudi Arabia; 10 Food Control Department, Faculty of Veterinary Medicine, Zagazig University, Zagazig 44519, Egypt; 11 Department of Microbiology, Faculty of Veterinary Medicine, Zagazig University, Zagazig 44511, Egypt

**Keywords:** thymol, thymoquinone, growth performance, digestive enzymes, antioxidant, autophagy, immunity, *Aeromonas sobria*

## Abstract

**Simple Summary:**

In modern aquaculture, fish have been subjected to intensive stressful conditions, which threaten their growth rate and increase their susceptibility to bacterial diseases. Innovative and sustainable production strategies in aquaculture have encouraged the utilization of plant-derived bioactive molecules (phytogenics) in fish diets, especially when they were used in a unique blend offering enumerable growth and health benefits. Therefore, our research was conducted to uncover the potential nutritional and immunological impacts of thymol (Thy) and/or thymoquinone (ThQ) with a disease shielding effect against *Aeromonas sobria*. Herein, the integration of dietary Thy and ThQ provided an efficient way to improve fish growth-related parameters and muscle antioxidant capacity. Moreover, we presented evidence for the unique immune-stimulating role of a Thy and ThQ combination with a successful forefront defense against *A. sobria* experimental infection in Nile tilapia. Our key findings motived the application of a dietary blend comprising Thy and ThQ as a functional feed that fights the challenges facing the aquaculture industry with maximized fish productivity.

**Abstract:**

Plant-derived bioactive compounds with promising nutritional and therapeutic attributes (phytogenics) are among the top priorities in the aquaculture sector. Therefore, the impact of thymol (Thy) and/or thymoquinone (ThQ) on the growth, immune response antioxidant capacity, and *Aeromonas sobria* (*A. sobria*) resistance of Nile tilapia was investigated. Four fish groups were fed a control diet and three basal diets supplemented with 200 mg/kg diet of Thy or ThQ and a blend of both Thy and ThQ at a level of 200 mg/kg diet each. At the end of the feeding trial (12 weeks), the tilapias were challenged intraperitoneally with virulent *A. sobria* (2.5 × 10^8^ CFU/mL) harboring aerolysin (*aero*) and hemolysin (*hly*) genes. The results revealed that tilapias fed diets fortified with a combination of Thy and ThQ displayed significantly enhanced growth rate and feed conversion ratio. Notably, the expression of the genes encoding digestive enzymes (pepsinogen, chymotrypsinogen, α-amylase and lipase) and muscle and intestinal antioxidant enzymes (glutathione peroxidase, catalase and superoxide dismutase) was significantly upregulated in Thy/ThQ-fed fish. An excessive inflammatory response was subsided more prominently in the group administrated Thy/ThQ as supported by the downregulation of *il-β*, *il-6* and *il-8* genes and in contrast, the upregulation of the anti-inflammatory *il-10* gene. Remarkably, dietary inclusion of Thy/ThQ augmented the expression of autophagy-related genes, whilst it downregulated that of *mtor* gene improving the autophagy process. Furthermore, Thy/ThQ protective effect against *A. sobria* was evidenced via downregulating the expression of its *aero* and *hly* virulence genes with higher fish survival rates. Overall, the current study encouraged the inclusion of Thy/ThQ in fish diets to boost their growth rates, promote digestive and antioxidant genes expression, improve their immune responses and provide defense against *A. sorbia* infections with great economic benefits.

## 1. Introduction

Aquaculture is considered one of the favorable food manufacturing sections and its sustainable growth is thought to be a potential strategy for maximizing the production of fish worldwide and enhancing human nutrition and food security [1,2]. Nile tilapia (*Oreochromis niloticus*) is one of the most cultured fish species with high global marketing values due to its high growth performance and resistance for stressful environmental conditions [3]. Recently, there has been an increase in the consumers’ preference for safe fish flesh with high quality and nutrition, which has an impact on the fish products’ market value and leads to the intensification of fish culture [1,2]. Improving fish growth, immunity and utilization of nutrients during intensive production systems are among their most important goals [4,5], which can be achieved via utilizing specific feed additives with growth-promoting, immunostimulant and antioxidant characteristics [3]. One of the main problems in the fish industry in developing countries is the overuse of antimicrobial agents in aquaculture, which results in the emergence of resistant microorganisms and the presence of antimicrobial residues in the fish flesh [6,7]. Furthermore, fish raised in intensive fish cultures are exposed to short-term food deprivation and high stocking density in response to overproduction [8], which makes them susceptible to stress conditions that have harmful effects on their physiological functions, and immune defense leading to significant economic losses and high mortality rates [9]. Fish have developed numerous strategies to combat oxidative stress via generating antioxidants those can remove dangerous free radicals with a consequence of preventing diseases and mitigating cell damage [1]. Pathogenic *Aeromonas* species (spp.) such as *Aeromonas hydrophila* (*A. hydrophila*) and *A. sobria* [10,11] have several strategies to disrupt the immune systems of fish resulting in the spread of serious diseases. The pathogenicity of these pathogens is complicated and they depend on many virulence factors such as invasion, adhesion, biofilm formation, motility, resistance to phagocytosis and production of exotoxins such as hemolysin/aerolysin, proteases, lipases and cytolytic toxins [4,8,12]. *A. sobria* infection in fish is fatal resulting in ulcer formation, ascites and hemorrhagic septicemia. The septicemia occurrence is mainly attributed to the production of two significant virulence genes; extracellular hemolysin (*hly*) and aerolysin (*aero*) [13].

Lately, there has been a fast development in bacteria resistant to the presently used antibiotics [14,15,16], because of the misuse of antibiotics in humans, animals and fish, especially in developing countries [17,18,19,20,21,22], that necessitate the usage of novel alternative antibiotics from medicinal plants to control the resistant strains [23,24]. Phytogenics are secondary metabolites of medicinal plants those are produced as a result of plants interaction with their surrounding environment. Phytogenics does not have any role in the plants main metabolic process; however, they potentially improve the ability of these plants to survive in adverse conditions [25]. Of note, phytogenics have growth-promoting, antibacterial, immunostimulant and anti-inflammatory characteristics by decreasing microbial loads and bacterial virulence genes expression and modifying anti- and pro-inflammatory cytokines-related genes expression [4,26,27,28]. These beneficial characteristics are attributed to the presence of multiple bioactive constituents such as essential oils (EOs), steroids, terpenoids, tannins, saponins, phenolics, glycosides, alkaloids and flavonoids [29]. Thus, they have been used recently as safe and effective natural replacements for antibiotics for the control and treatment of various diseases [30,31]. Recently, several studies have utilized eco-friendly natural feed supplements, which promote the health and growth of fish, ameliorate the negative effects of bacterial infections, especially *Aeromonas* spp. during intensive fish culture, and provide consumers with high-quality fish meat [13,32,33]. Of note, the phytogenics′ modes of action could vary because of the various sources and forms of the utilized active constituents [26]. Phytogenics can be classified according to their appearance and physical activities into EOs, crude, mixtures of plant extracts and the plants processed parts [29]. Recently, the feed industry has categorized the majority of phytogenics either as feed additives or feed ingredients. Thus, EOs, spices, herbs, and non-volatile plant extracts are commonly used as feed additives. Plant extracts and EOs are classified as ‘sensory additives’ by European legislation [29,34]. Meanwhile, unprocessed herbs are categorized as feed ingredients; thus, they do not require any authorization before utilization in the feed preparation [29,35].

Among the phytogenic feed supplements utilized in aquaculture are thymol (Thy) and thymoquinone (ThQ), which are considered the main phenolic components of thyme (*Thymus vulgaris*) and black cumin (*Nigella sativa*) EOs, respectively. They have been utilized in the food and aquaculture industry for their medicinal characteristics since ancient times [36,37,38,39]. Additionally, they could be utilized as feed supplements in fish nutrition because they have potential advantageous and therapeutic efficacy on meat quality, antioxidant potential, immunity, nutrient utilization, and growth performance [36,39]. These beneficial functions are thought to be achieved through improving the mucosal barriers and gastrointestinal integrity, which leads to enhancement of the digestibility and minimizes the interaction between dangerous components/free radicals and cellular biological components [1,4,36,40,41]. Several researches have reported the beneficial effects of dietary Thy and ThQ on the growth performance, immunological, biochemical and hematological variables of various fish species including Nile tilapia (*O. niloticus*) [42,43,44], rohu (*Labeo rohita*) [39], common carp (*Cyprinus carpio*) [45], rainbow trout (*Oncorhynchus mykiss*) [46,47,48], Asian sea bass (*Lates calcarifer*) [49], climbing perch (*Anabas testudineus*) [50] and channel catfish (*Ictalurus punctatus*) [51]. 

Few pieces of researches have studied the effect of Thy and ThQ on the transcription of genes encoding digestive enzymes and antioxidant potential in Nile tilapia. However, to the best of our knowledge, there has been no researches describing the in vivo effects of Thy and ThQ on the expression levels of autophagy-related genes and *A. sobria* resistance of Nile tilapia. Therefore, the current work aimed to study, for the first time, the in vivo effectiveness of Thy and/or ThQ on Nile tilapia growth performance, digestive and antioxidant enzymes, cytokines and autophagy-related genes expression as well as *A. sobria* resistance in Nile tilapia post-challenge with virulent *A. sobria* strain.

## 2. Materials and Methods

### 2.1. Thymol and Thymoquinone

The thymol and thymoquinone utilized in our study were obtained from Sigma-Aldrich (St. Louis, MO, USA). Thymol and thymoquinone are the principle phenolic components of thyme (*Thymus vulgaris*) and black cumin (*Nigella sativa*) EOs, respectively.

### 2.2. Rearing Conditions of *Nile tilapia*

In total, 400 healthy Nile tilapia (*O. niloticus*) were purchased from the Central Laboratory for Aquaculture Research, Abbassa, Abu-Hammad, Sharkia, Egypt. On arrival, the fish were acclimatized for two weeks before starting the experiment in glass aquaria supplied with dechlorinated water and continuous aeration through air compressors, and they were offered a basal diet via hand feeding. During the adaptation and whole experimental periods, the water temperature was 28 ± 2 °C, the dissolved oxygen was adjusted via an oxygen meter (YSI Company model 56, Yellow Springs, OH, USA) to be 5.5 ± 0.4 mg/L, the pH was measured by a pH meter (Orion; Abilene, TX, USA) to be 7.5, the nitrate was 5.7 mg/L, the nitrite was 0.038 mg/L and the ammonium was 0.27 mg/L. After the adaptation period, the tilapias were weighed individually (initial body weight = 12.6 ± 0.2 g) and randomly assigned into four equal groups (five replicates each; 20 fish/replicate).

### 2.3. Experimental Design, Diets and Feeding Trial

The experimental groups consisted of G1 (tilapia fed basal diets with thymol supplementation at a concentration of 200 mg/kg diet), G2 (tilapia fed basal diets with thymoquinone supplementation at a concentration of 200 mg/kg diet), G3 (tilapia fed a basal diets with thymol and thymoquinone mixture (1:1) supplementation at a concentration of 200 mg/kg diet each) and the control (tilapia fed a basal diet without any supplementations). The fish were offered three isonitrogenous (crude protein, 35%) and isocaloric (digestible energy, 2900 kcal) experimental diets for 12 weeks of the feeding trial. Nutrient constituents of the control basal diet were formulated following the Nile tilapia nutrient recommendations of the Natural Research Council [52] as presented in Table 1. Separate dietary ingredients were grounded, weighed according to the diet formula, thoroughly mixed, and manufactured into pellets via a pelleting machine. The EOs were sprayed uniformly over the feed according to the diet formula. The diets were air dried, stored in air-sealed plastic bags and kept at 5 °C until used. The basal diet chemical composition was verified via the standard analysis procedure [53]. The fish were fed twice per day (9:00 and 16:00 h) for 12 weeks until apparent visual satiation. All the glass aquaria were supplied with a large air generator pump and dechlorinated water. Settled uneaten feed, wastes from the fish and 3/4 of the water of the aquaria were eliminated daily and replaced with well-aerated and clean water.

### 2.4. Evaluating Growth Performance-Related Parameters and Profitability

Tilapias in G1, G2, G3 and control groups were weighed separately to determine the final body weight, and final weight gain. After that, we calculated the growth performance and profitability related parameters as described elsewhere [1,40,54] as following: final weight gain (g) = final weight (g) − initial weight (g), specific growth rate = 100 [Ln final weight (g) − Ln initial weight (g)]/experimental period (day), feed intake = the diets consumed by the fish over the experimental period/number of live fish, feed cost = total feed intake/fish × price of 1 kg feed, feed conversion ratio = feed intake/final weight gain, protein efficiency ratio = fish weight gain (g)/protein intake (g), feed cost/kg gain (USD/fish) = capital turnover (CTO) = selling costs/ total costs, net profit (USD/fish) = selling costs − total costs, economic efficiency = net profit/total feed cost, fish survival (%) = 100 × (fish number after the experiment/fish number at the beginning of the experiment), profit loss = (total return × no. of dead tilapias) − (total tilapias’ costs, which reared till death × no. of dead tilapias), cost of mortality = no. of dead tilapias − (average tilapias’ cost, which reared till death + average fixed cost), and the mortality losses are the sum of profit loss and cost of mortality. 

### 2.5. Sampling and Anlalysis Techniques

#### 2.5.1. Blood and Tissue Sampling

At the end of the feeding trail, five fish from each replicate were trapped randomly for the collection of blood from their caudal veins. The obtained blood samples were transmitted directly into Eppendorf plastic tubes with an anticoagulant for assessing the hematological parameters or permitted to coagulate for evaluating biochemical and immunological markers. Centrifugation of the coagulated samples at 3000 rpm for 20 min was carried out for separation of the serum, which was last collected and deposited at −20 °C until use. After cervical amputation, muscle (dorsal region), splenic, intestinal and hepatopancreatic tissues were collected instantly, rinsed with normal saline and quickly kept at −80 °C until further gene expression analysis.

#### 2.5.2. Blood Hematological and Serum Biochemical and Immune-Related Indices

Red blood cells (RBCs) counts were estimated by a means of Neubauer hemocytometer (Sigma-Aldrich, Darmstadt, Germany) and Natt-Herrick solution, and the hemoglobin (Hb) level was calculated utilizing the Hb colorimetric procedure. Hematocrit (Ht) was measured in the partial heparinized whole blood by the microhematocrit method via centrifuging the samples at 13,000 rpm for 5 min [55]. The serum biochemical profile [cholesterol, triacylglycerol, aspartate transaminase (AST), alanine transaminase (ALT), urea, and creatinine] was determined spectrophotometrically using commercial analysis kits (Spinreact, Ctra. Santa Coloma, Spain) following the manufacturer’s guidance. The serum lysozyme activity and immunoglobulin M (IgM) level were detected through the agarose gel cell lysis technique and ELISA Kit, Catalog No. CSB-E12045Fh, respectively as previously described [4]. Moreover, the activities of serum myeloperoxidase (MPO) and alternative complement were detected following the approaches described previously [56,57].

#### 2.5.3. Quantitative Reverse Transcription Polymerase Chain Reaction Procedures for Gene Expression Analysis

Assessing the expression of the genes encoding digestive enzymes (pepsinogen, chymotrypsinogen, α-amylase and lipase), cytokines [tumor necrosis factor-α (*tnf-α*), interleukin (*il*)*-10*, *il-8*, and *il-β*], antioxidant enzymes [superoxide dismutase (*sod*), catalase (*cat*), and glutathione peroxidase (*gsh-px*)], autophagy (*atg5* and *atg12*), microtubule-associated proteins 1A/1B light chain (*lc3-II*), beclin-1 (*bcln-1*) and the mechanistic target of rapamycin (*mtor*) in fish muscle, intestinal, splenic and hepatopancreatic tissues were detected through a quantitative reverse transcription polymerase chain reaction (qRT-PCR) technique. The sequences of primers targeting the investigated genes are presented in Table 2. Extraction of RNA was carried out through an RNeasy Mini Kit (Qiagen, Hilden, Germany) as per the manufacturer’s direction and then the quantity and purity of the extracted RNA were detected via Nano-Drop ND-1000 Spectrophotometer (NanoDrop Technologies, Wilmington, NC, USA). The analysis of relative expression levels of the examined genes was determined, in a triplicate, in Stratagene MX3005P real-time PCR machine (Stratagene, La Jolla, CA, USA) utilizing QuantiTect SYBR Green Kit (Qiagen, Hilden, Germany). The 2^−ΔΔCT^ procedure was utilized for analysis of the relative mRNA expression levels [58], where the β-actin gene was the normalizing agent (endogenous control). The results were expressed as fold changes in the transcriptional levels of the target genes in the tested specimens concerning those in the control group. Melting curve analysis was carried out at the termination of the amplification cycles to certify that all reactions amplified the correct PCR products [4].

### 2.6. Aeromonas sobria Challenge and Expression Analysis of Its Virulence-Related Genes

A presumptive *A. sobria* virulent strain isolated from diseased Nile tilapia during a previous microbiological investigation was utilized for the challenge test. The used strain was identified via the Vitek^®^ 2 system (bioMérieux, St. Louis, MO, USA) and a PCR-restriction fragment length polymorphism examination of the *16S rRNA* gene according to the protocols described previously [13,59]. Moreover, the strain was verified to be virulent through PCR screening for the presence of genes encoding hemolysin and aerolysin using primers and PCR cycling conditions detailed formerly [56,59,60,61]. Enrichment of the used bacterial strain was carried out in brain heart infusion broth (Oxoid, Hampshire, UK) to be passed twice in healthy Nile tilapia for enhancing its pathogenicity and then it was re-isolated from sacrificed or freshly dead tilapias and utilized for the challenge study. It was confirmed that the tilapias were free from *A. sobria* infection before starting the challenge trial. To assess the in vivo efficacy of supplementing Thy and/or ThQ for 12 weeks in a fish diet, ten healthy fish from each replicate were maintained in their aquaria, and injected intraperitoneally with 0.2 mL of *A. sobria* broth culture (2.5 × 10^8^ CFU/mL), which was detected through a previous lethal dose 50% (LD_50_) trial [1,62]. Post challenge, all experimental fish were monitored for observing any abnormal clinical signs and recording the mortality rates was then carried out. Moreover, clinically diseased, moribund and recently dead fish were aseptically subjected for re-isolation and identification of the challenging bacterial strain.

At 7 and 14 days post-challenge, splenic tissues were obtained from five tilapias per experimental group to discover the impact of Thy and/or ThQ on the expression levels of *A. sobria aero* and *hly* virulence genes using *16s rRNA* as an internal control gene. The detailed description for the qRT-PCR procedures has been mentioned above (Section 2.5.3).

### 2.7. Statistical Analysis

The resulting data were exposed to one-way ANOVA analysis for evaluating the impact of dietary Thy and/or ThQ supplementation. Variations among means were assessed at a 5% probability level utilizing Tukey as a post-hoc test. All the statistical assessments were performed through SPSS program version 20 (SPSS, Richmond, VA, USA).

## 3. Results

### 3.1. Fish Growth Performance and Profitability Variables

The significant (*p* < 0.05) beneficial activities of dietary Thy and/or ThQ were observed in the fish growth performance variables (Table 3). The most significant (*p* < 0.05) enhancements in the protein efficiency ratio, final weight gain and final body weight were detected in the Thy and ThQ mixture group. Additionally, supplementation of Thy and/or ThQ had no significant effect (*p* > 0.05) on fish total feed intake. The greatest improvement in the specific growth rate and feed conversion ratio were noticed in the fish received diets enriched with the Thy and ThQ mixture. Notably, net profit, and economic efficiency were significantly (*p* < 0.05) decreased in the control group when compared with the other Thy and/or ThQ experimental groups (Table 3). 

### 3.2. Blood Hematological and Serum Biochemical and Immunological Indicators

The hematological, biochemical and immunological characteristics of the fish after a 12-week feeding trial are shown in Table 4. Inclusion of Thy and/or ThQ had no significant (*p* > 0.05) impact on hematological (RBCs; Hb and Ht), liver (ALT and AST), and kidney (urea and creatinine) function tests. Moreover, the most greatly (*p* < 0.05) reduced cholesterol level was noticed in the fish fed diets enriched with a combination of Thy and ThQ. Compared to the control group, supplementing fish diets with Thy and ThQ mixture significantly (*p* < 0.05) reduced the level of triacylglycerol. Concerning the immunological parameters of the fish, lysozyme, MPO and IgM reached their significant (*p* < 0.05) peaks post dietary supplementation of Thy and ThQ mixture. Additionally, the highest serum alternative complementary level was observed in the Thy/ThQ supplemented group.

### 3.3. Gene Expression Profiles of Digestive and Antioxidant Enzymes

**Figure 1:**
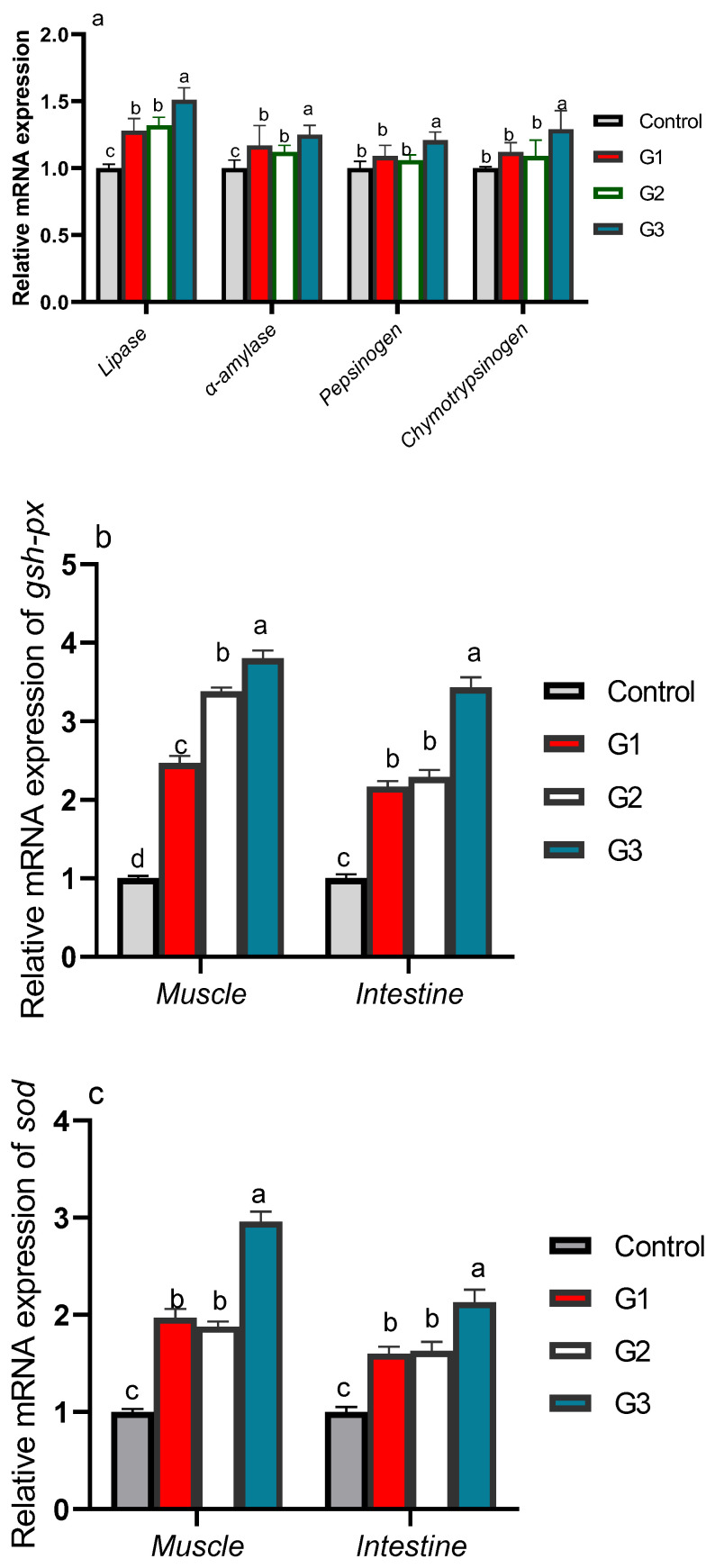

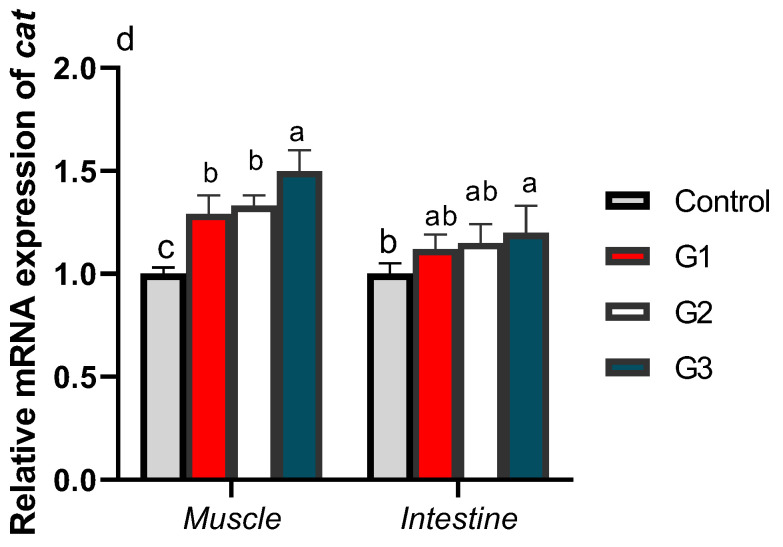
Transcriptional levels of digestive enzymes (lipase, α-amylase, pepsinogen and chymotrypsinogen) encoding genes (**a**) and muscle and intestinal antioxidant-related genes [*gsh-px*; glutathione peroxidase (**b**), *sod*; superoxide dismutase (**c**), and *cat*; catalase (**d**)] in fish fed diets fortified with Thy and/or ThQ. Results are expressed as means ± SEM. Columns with various letters represent significant variations (*p* < 0.05). Control: tilapia fed basal diets without any supplementations, G1: tilapia fed basal diets with thymol (Thy) supplementation at a concentration of 200 mg/kg diet, G2: tilapia fed basal diets with thymoquinone (ThQ) supplementation at a concentration of 200 mg/kg diet, and G3: tilapia fed basal diets with Thy/ThQ supplementation at a concentration of 200 mg/kg diet each.

The gene expression patterns of digestive and antioxidant enzymes of the fish fed diets supplemented with Thy and/or ThQ are illustrated in Figure 1. Feeding fish on Thy/ThQ exhibited the most noticeable (*p* < 0.05) expression levels of pepsinogen, chymotrypsinogen, α-amylase and lipase-related genes (Figure 1a). Notably, dietary fortification of Thy and/or ThQ promoted the expression levels of antioxidant encoding genes (Figure 1b–d). The highest significant (*p* < 0.05) mRNA expression level of muscle *gsh-px* gene was detected in the Thy/ThQ enriched group (Figure 1b). Moreover, the relative *sod* and *cat* expression levels in fish muscle were significantly (*p <* 0.05) upregulated in tilapias fed a combination of Thy and ThQ, followed by those fed Thy or ThQ alone unlike the control group (Figure 1c,d). Generally, the expression levels of antioxidant encoding genes concerning dietary inclusion of Thy and/or ThQ were noticed to be higher in the fish muscle than in the fish intestine. The transcriptional levels of intestinal *gsh-px*, *sod* and *cat* genes were maximized in fish fed a combination of Thy and ThQ (Figure 1b–d).

### 3.4. Relative mRNA Expression of Cytokines and Autophagy-Related Genes 

**Figure 2:**
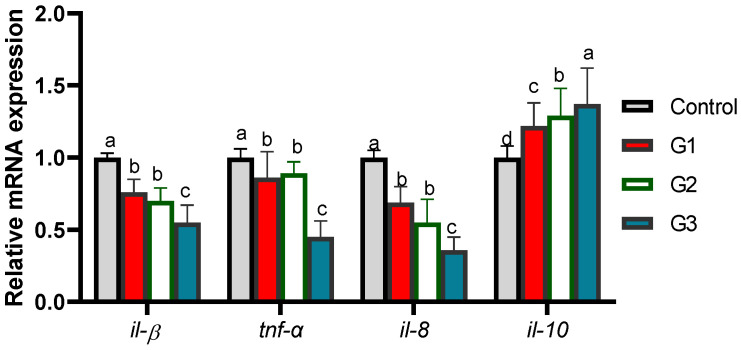
Transcriptional levels of cytokines’ genes [Interleukin (*il*)-*β*, *il-8*, *il-10*, and tumor necrosis factor-α (*tnf-α*)] in fish fed diets fortified with Thy and/or ThQ. Results are expressed as means ± SEM. Columns with various letters represent significant variations (*p* < 0.05). Control: tilapia fed basal diets without any supplementations, G1: tilapia fed basal diets with thymol (Thy) supplementation at a concentration of 200 mg/kg diet, G2: tilapia fed basal diets with thymoquinone (ThQ) supplementation at a concentration of 200 mg/kg diet, and G3: tilapia fed basal diets with Thy/ThQ supplementation at a concentration of 200 mg/kg, diet each.

The efficacy of the dietary inclusion of Thy and/or ThQ on the cytokines encoding genes expression are shown in Figure 2. Of note, the transcriptional levels of *il-B*, *tnf-α* and *il-8* genes were likely to be significantly (*p* < 0.05) decreased after supplementing Thy and ThQ in combination, followed by their inclusion singly unlike the control group. Meanwhile, the maximum significant (*p* < 0.05) expression level of *il-10* gene was observed in the Thy/ThQ supplemented group.

**Figure 3:**
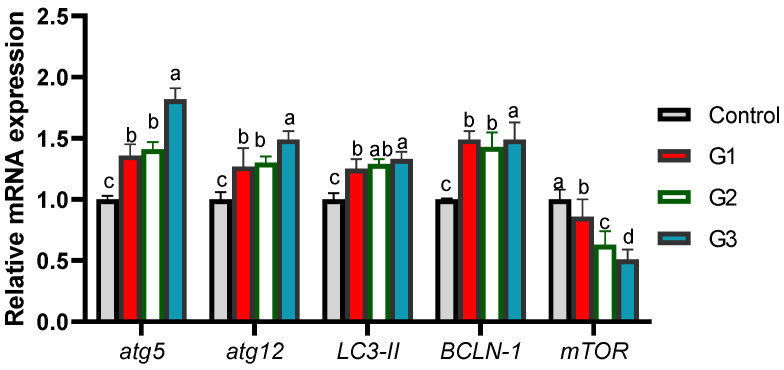
Analysis of quantitative reverse transcription polymerase chain reaction of autophagy (*atg5* and *atg12*), mechanistic target of rapamycin (*mtor*), beclin-1 (*bclN-1*), and microtubule-associated proteins 1A/1B light chain (*lc3-II*) encoding genes in fish fed diets fortified with Thy and/or ThQ. Results are expressed as means ± SEM. Columns with various letters represent significant variations (*p* < 0.05). Control: tilapia fed basal diets without any supplementations, G1: tilapia fed basal diets with thymol (Thy) supplementation at a concentration of 200 mg/kg diet, G2: tilapia fed basal diets with thymoquinone (ThQ) supplementation at a concentration of 200 mg/kg diet and G3: tilapia fed basal diets with Thy/ThQ supplementation at a concentration of 200 mg/kg diet each.

The efficacy of supplementing Thy and/or ThQ on the expression of autophagy encoding genes is illustrated in Figure 3. It was remarkable that administration of Thy and ThQ mixture in diet of the fish had the most prominent (*p* < 0.05) downregulating role on *mtor* expression. In contrast, fish fed a combination of Thy and ThQ displayed the highest (*p* < 0.05) expression levels of *atg5*, *atg12* and *bclN-1* genes. Additionally, all groups received Thy and/or ThQ displayed substantial (*p* < 0.05) elevations in the transcriptional levels of the *lc3-II* gene.

### 3.5. Effect of Diets Fortified with Thy and/or ThQ on Survival Percentages and Relative Expression of A. sobria Virulence-Related Genes

**Figure 4:**
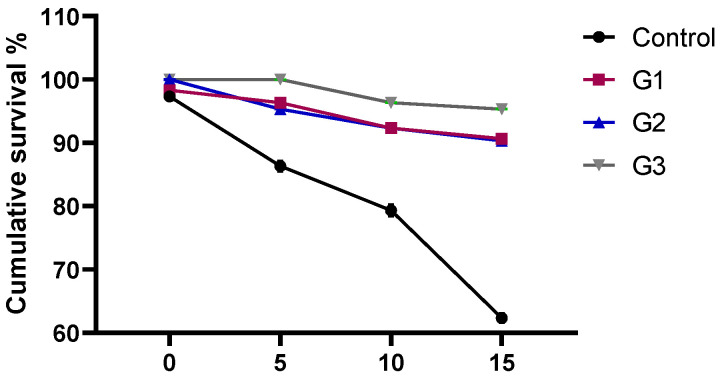
Effect of diets fortified with Thy and/or ThQ on cumulative survival percentage of Nile tilapia challenged with *Aeromonas sorbia*. Control: tilapia fed basal diets without any supplementations, G1: tilapia fed basal diets with thymol (Thy) supplementation at a concentration of 200 mg/kg diet, G2: tilapia fed basal diets with thymoquinone (ThQ) supplementation at a concentration of 200 mg/kg, diet and G3: tilapia fed basal diets with Thy/ThQ supplementation at a concentration of 200 mg/kg diet each.

**Figure 5:**
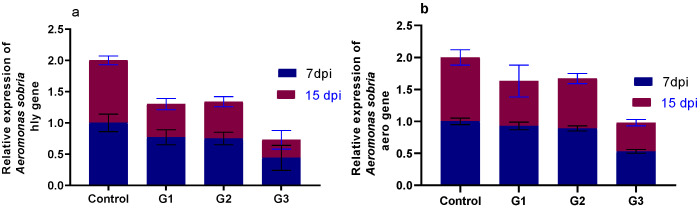
Effect of diets fortified with Thy and/or ThQ at 7 and 15 days post challenge with *Aeromonas sobria* on relative mRNA expression levels of *Aeromonas sobria* virulence genes; *hly* (**a**) and *aero* (**b**). Results are expressed as means ± SEM. Control: tilapia fed basal diets without any supplementations, G1: tilapia fed basal diets with thymol (Thy) supplementation at a concentration of 200 mg/kg diet, G2: tilapia fed basal diets with thymoquinone (ThQ) supplementation at a concentration of 200 mg/kg diet, G3: tilapia fed basal diets with Thy/ThQ supplementation at a concentration of 200 mg/kg diet each, and dpi: days post infection.

The percentage of cumulative survival varied from 62 % to 95%, with the highest survival rate after *A. sobria* challenge identified in the fish fed a dietary combination of Thy and ThQ (Figure 4). The relative expression levels of *A. sobria hly* and *aero* virulence genes are displayed in Figure 5. The results considering qRT-PCR analysis at 7 and 15 days post challenge with *A. sobria* showed that feeding on Thy/ThQ significantly (*p* < 0.05) decreased the expression levels of *aero* and *hly* virulence genes unlike the control non-supplemented group. Notably, 15 days post *A. sobria* challenge, the inclusion of the Thy and ThQ mixture exhibited prominent decreasing influence on the expression of the investigated virulence genes.

## 4. Discussion

Recently, medicinal plants, especially phytophenolic compounds of EOs, have been strongly investigated for their useful alternative effects on the health of humans, fish and animals in comparison to chemicals [1,4]. Using eco-friendly feed additives from herbal medicine to improve the utilization of nutrients and animal performance become widely accepted [4]. Application of phytogenic active compounds have beneficial outcomes in enhancing the intensively cultured fish growth, profitability and diseases resistance via regulating their immune systems and digestibility, which subsequently improve their productivity [36,37,38]. The cost-effectiveness and biological efficacy of feed additives should be taken into account when choosing whether to use them in animal feeding. Considering thymol and thymoquinone as efficient bioactive compounds, little researches have investigated their perspective roles on Nile tilapia growth, immunity, and antioxidant potential. Additionally, to the best of our knowledge, no data have been consolidated on the potential effects of thymol and thymoquinone on the transcriptional levels of digestive enzymes and autophagy-related genes and *A. sobria* resistance of Nile tilapia. 

In the current study, inclusion of Thy and ThQ mixture had the most prominent role in inducing higher growth performance variables and improving the feed utilization efficiency with great net profit and economic efficiency of Nile tilapia unlike the control or using each compound individually. In accordance with our findings, the combination of various phytogenic compounds (limonene, thymol, cinnamon and oregano) had high growth promoting effects in Nile tilapia [63,64,65]. In agreement with our findings, previous studies stated that EOs mixture or thymol had improved the body weight, body weight gain, feed conversion ratio with more net revenue and feed economic efficiency in Nile tilapia [43,44,66] and rainbow trout [40,47]. Moreover, an increased final body gain (up to 44%) and superior FCR were noticed in channel catfish fed a combination of carvacrol, limonene, thymol, and anethol compounds [67]. Additionally, previous studies stated that dietary inclusion of thymoquinone enhanced the growth parameters at the levels of 2.5% in Rohu [39], 5 g/kg in Asian sea bass [49], climbing perch [50] and channel catfish [51]. The remarkable higher growth performance of Nile tilapia following feeding on Thy and ThQ mixture might be explained by their roles in sustaining the structure and boosting the functions of digestive system as previously documented [36,68]. In line with the improved growth performance in Thy and ThQ mixture group, the expression levels of digestive enzymes (pepsinogen, chymotrypsinogen, α-amylase and lipase) encoding genes were also provoked. Digestive enzymes synthesis can be modified by genes, hormones and nutritional aids [67] and their higher expression levels reflect the important function of specific dietary feed additives or even their adaptations by animals [69,70]. Herein, the upregulated expression of pepsinogen, chymotrypsinogen, α-amylase and lipase genes after feeding on Thy and ThQ mixture indicated their promising roles in enhancing the formation of such digestive enzymes, which in turn have a positive influence on the digestive process and feed utilization. In agreement with our results, a recent study stated that addition of EOs (3 g/kg) to the diet of Nile tilapia significantly enhanced growth performance variables, utilization of feed, the digestibility and the activity of lipase enzyme [61]. The variations in the EOs effects among different studies could be attributed to the variances in their supplementation levels, constituents, extractions methods and sources. 

The fish antioxidant defense system is greatly associated with their immune and health conditions. Situations of diseases lead to physiological changes associated with oxidative stress, which results from a disproportion in the generation of nitrogen species/reactive oxygen species (ROS) and antioxidant and immunological reactions [8,10,71]. The antioxidant enzymes are important regulators of the antioxidant defense system of fish through eliminating the excessive ROS and thus, maintaining the homeostasis of cells [4]. Previous studies stated that the utilization of immunostimulant-enriched food could stimulate the fish antioxidant defense system [72]. The current work described that the relative *gsh-px*, *sod* and *cat* expression levels in fish muscle were significantly (*p* < 0.05) increased in fish fed a combination of Thy and ThQ indicating the high capacity of Thy/ThQ against oxidative damage. These results are in agreement with the findings of recent studies, which reported that dietary inclusion of thymol to Nile tilapia increased the expression levels of *cat* [43,44,68] and *sod* [43] genes suggesting the antioxidant characteristics of thymol. In consistence with our findings, a recent study stated that dietary inclusion of black cumin at a concentration of 30 g/kg of diet in Nile tilapia elevated the expression levels of *sod* and *gsh-px* [42] genes signifying the antioxidant characteristics of thymoquinone. Additionally, a recent study stated that dietary inclusion of black seed at the level of 2.5% prominently increased the levels of catalase, and glutathione peroxidase in rohu fish [39]. In the current study, the differences in the antioxidant levels in the investigated tissues suggested that Thy and ThQ had tissue-specific antioxidant activities. Increasing expression levels of antioxidant enzymes encoding genes were more prevalent in the hepatic tissues, followed by the muscular and intestinal tissues, since the liver is the main organ used in metabolic processes and the eradication of dangerous constituents [73]. Upregulation of genes encoding antioxidant enzymes in fish flesh after feeding EOs resulted in increased activities of oxidative enzymes, which prevent the spoilage of fish products [4,42,43].

Blood immunological and biochemical parameters are critical indicators of the fish general health. Notably, bacterial infections cause systemic inflammatory responses, which are significant problems in generating stresses on the immune system that harm the fish performance and threaten their general health [1]. Herein, the inclusion of Thy and/or ThQ had no significant impact (*p* > 0.05) on hematological (RBCs, Hb and Ht), liver (ALT and AST), and kidney (urea and creatinine) function tests. Our results are similar to the findings of a recent study, which stated that dietary supplementation of 1% thyme oil in Nile tilapia had no significant effects on blood biochemical parameters such as total protein, albumin, globulin, ALT and AST [74]. Interestingly, the extracts of EOs could positively modulate the immune response of fish, which may be involved in enhancing their resistance to microbial infections. Lyzozyme is the immune system’s non-specific defense component, which is utilized to investigate the capability of the immune system in fish to overcome pathogenic infections [1,26,75]. Moreover, the immunoglobulins such as IgM are considered one of the most fundamental indicators of fish immunity. They were tested after feeding fish with basal diets supplemented with EOs and improvements in their levels may be a result of the strong innate immune responses [1,4]. In the current work, lysozyme, MPO and IgM reached their significant peaks (*p* < 0.05) post dietary supplementation of the Thy and ThQ mixture. These results are in agreement with the findings of previous studies, which stated that dietary inclusion of EO and thymol (2 mL/kg diet) increased the levels of LYZ and IgM in Nile tilapia [43,44] and rainbow trout [48] because they had immunostimulant activities via their beneficial effects on the humoral non-specific immune response. Moreover, a recent study reported that dietary supplementation of black cumin had increased the LYZ, MPO and IgM levels in Nile tilapia [42] and rainbow trout [46]. In line with the upregulation of the expression levels of digestive enzymes encoding genes (pepsinogen, chymotrypsinogen, α-amylase and lipase), supplementing fish diets with Thy/ThQ improved the triacylglycerol level and lowered the cholesterol level compared to the control group. This is consistent with a recent study, where EOs treatment attenuated the cholesterol level and enhanced the triacylglycerol level in fish [4]. Cytokines have fundamental regulatory activities on the inflammatory reactions of the gastrointestinal tract (GIT). Following microbial invasion, cytokines are secreted by the GIT’s immune cells because of their important activities in the host immune defense against microbial infection [31]. Balancing between pro- and anti-inflammatory cytokines is important for sustaining their functions in fish. Inflammatory reactions have a fundamental role in the innate immune defense against bacterial infections [1,4]. The *il-β*, *tnf-α* and *il-8* are important pro-inflammatory cytokines in fish, which are involved in the immune defense against microbial infections [76] through enhancing phagocytosis, the production of lysozyme and bactericidal activities [77], but *il-10* has effective anti-inflammatory activities [78]. In line with the increased levels of LYZ, MPO and IgM in the Thy/ThQ group, the transcriptional levels of *il-B*, *tnf-α* and *il-8* genes were significantly decreased after supplementing Thy and ThQ mixture to Nile tilapia reflecting their immunostimulant characteristics. These immunostimulant characteristics might be attributed to the role of EOs in improving the non-specific immune system of the host via the nonspecific killing of microorganisms and tumor cells [26]. Similarly, a previous study stated that thymoquinone decreased the expression level of *il-B* gene in Nile tilapia post challenge with *A. hydrophila* [79]. Herein, Thy and ThQ mixture significantly upregulated the expression level of *il-10* gene in Nile tilapia indicating their anti-inflammatory activities. A similar increase in *il-10* gene expression level was detected in Nile tilapia fed thymoquinone post challenge with *Burkholderia cepacia* [42]. Autophagy is a mechanism, in which lysosomes used by the cells to destroy organelles and macromolecules are damaged. The innate immune response and autophagy might act through a combined signaling pathway [8]. To control the adaptive immunity, the immune system uses the destroyed products from the autophagy process [80]. The autophagy incidence is attributed to the contribution of a unique series of autophagy-related proteins such as atg12-atg5, which have significant effects on the induction, expansion, nucleation, maturation, termination and degradation of autophagosomes [81]. Atg5 is an autophagic protein that has a significant effect on the development of autophagic vacuoles, thus it is fairly well conserved across most eukaryotes [82]. The LC3 abundance is related to the number of autophagic vacuoles, thus it is considered an autophagic marker [83]. The *bclN-1* gene is a target gene correlated to autophagy in mammalian cells and it is considered a homolog of yeast *atg6* and it is concerned with the formation of autophagosomes. Additionally, *bclN-1* has a significant role in the formation of tumors by controlling the autophagic activity [84]. Furthermore, *mtoR*, a serine/threonine kinase, has a main role in the autophagy pathway and cell metabolism and there is a reverse relation between the activation of *mtoR* and initiation of autophagy [85]. In the current study, fish fed a combination of Thy and ThQ displayed the highest expression levels of *atg5*, *atg12*, *lc3-II* and *bclN-1* genes and the lowest downregulating level of *mtoR* expression. In agreement with our results, a previous study stated that natural feed supplements upregulated *atg5*, *atg12*, *lc3-II* and *bclN-1* genes and downregulated the *mtor* gene in Nile tilapia post *A. hydrophila* challenge [8]; however, there are no studies investigating the effect of thymol and thymoquinone on autophagy-related genes in Nile tilapia.

Nutritional immunology is a novel method for preventing diseases in aquaculture through utilizing nutrients to overcome difficulties of vaccination programs [8]. Additionally, enhancement of fish feeding and veterinary management for disease prevention will enhance the economic and productive efficiency of fish farms. Herein, the maximum survival percentage after *A. sobria* challenge was identified in fish fed a dietary combination of Thy and ThQ. At 7 and 15 days post challenge with *A. sobria*, dietary inclusion of Thy/ThQ significantly downregulated the expression levels of *aero* and *hly* virulence genes concerning the non-supplemented control group. In agreement with our results, a recent study stated that dietary inclusion of clove oil decreased mortality rates and downregulated the transcription of *hly* and *aero* virulence genes following *A. sobria* challenge in catfish [13]. Additionally, previous studies showed that dietary inclusion of thymoquinone increased the survivability and decreased the mortality of common carp [45] and climbing perch [50] following *Pseudomonas fluorescens* and *A. hydrophila* challenge, respectively. Moreover, a previous study reported that dietary inclusion of thymol decreased the mortality rates in channel catfish following *A. hydrophila* challenge [51]. Similarly, a recent study reported that inclusion of natural eco-friendly feed supplements decreased the mortality rates and downregulated the virulence genes of *A. hydrophila* in Nile tilapia post challenge with *A. hydrophila* strain [8] suggesting their antibacterial activities; however, to the best of our knowledge, there are no studies investigating the effects of thymol and thymoquinone on survival percentages and relative expression of *A. sobria* virulence-related genes in Nile tilapia post challenge with *A. sobria* strain. The decreased mortality rate in fish fed the Thy/ThQ mixture post challenge with *A. sobria* could be explained by their strong immunostimulant roles, which in turn reduced the undesirable effects of bacterial infection. Moreover, the downregulating role of Thy/ThQ mixture might be attributed to the inhibition of quorum sensing, which is the bacterial gene regulation system that coordinates the expression of several virulence factors [86].

## 5. Conclusions

Our findings concluded how single or combined Thy/ThQ based diets positively affected performance, immunity and diseases resistance in Nile tilapia. Herein, molecular data considering digestive, antioxidants and immunity provided a better tool for understanding the mode of actions of Thy/ThQ that supported their beneficial functions. Post challenge, administration of Thy/ThQ offered new updated information considering their downregulating efficacy on the expression of *aero* and *hly* virulence genes of *A. sobria* for optimizing the output of defense against its infection. Finally, the beneficial properties of Thy/ThQ integration have opened up numerous avenues for their application as new dietary supplements.

## Figures and Tables

**Table 1:** The basal diet ingredients and chemical composition.

Ingredient	%
Fish meal	27
Yellow corn	27.5
Soybean meal	28.5
Rice bran	9
Corn gluten	2.5
Lysine	0.1
DL-Methionine, 98%	0.2
Fish oil	2.8
Di-calcium phosphate	0.3
Threonine	0.1
* Vitamins and minerals premix	2
**Chemical analysis**	
Digestible energy (kcal/kg)	2922
Nitrogen free extract %	42.06
Methionine, %	0.93
Ether extract, %	5.17
Crude protein, %	35.00
Lysine, %	2.33
Available P, %	0.58
Ca, %	1.05

* Vitamins and minerals/kg of product: 30 mg cobalt, 140 mg biotin, 220 mg folic acid, 2800 mg copper, 4000 mg pantothenic acid, 0.63 g antioxidant, 750 mg manganese, 65 mg selenium, 835 mg iron, 17,300 mg zinc, 110 mg iodine, 5000 mg niacin, 2465 mg vitamin B6, 2,000,000 IU vitamin A, 1300 mg vitamin B1, 2200 mg vitamin B2, 3750 mg vitamin B12, 500 mg vitamin K, 10,000 IU vitamin E, 300,000 IU vitamin D3, and 28,000 mg vitamin C.

**Table 2:** Primers’ sequences employed for quantitative reverse transcription polymerase chain reaction assay.

Target Gene	Primer Sequence (5′-3′)	Accession No. (GeneBank/Ensemble)
*β-actin*	F-TGGCATCACACCTTCTATAACGA R-TGGCAGGAGTGTTGAAGGTCT	XM_003455949.2
Pepsinogen	F-TGACCAATGACGCTGACTTG R-GGAGGAACCGGTGTCAAAAATG	JQ043215.1
Chymotrypsinogen	F-TTCTGCCTTCGCTTCTCATC R-TTCAACGCCATCTGCTACTG	ENSONIG00000003237
Lipase	F-TCGGTGGATGGCATGATGGAGA R-GCGACTGGATAGTGCTGCTGAG	ENSONIG00000005832
α-amylase	F-GCGACTGGATAGTGCTGCTGAG R-TGGCGTTGGGCTGACATTGC	ENSONIG00000018530
*gsh-px*	F-CCAAGAGAACTGCAAGAACGA R-CAGGACACGTCATTCCTACAC	NM_001279711.1
*cat*	F-TCAGCACAGAAGACACAGACA R-GACCATTCCTCCACTCCAGAT	XM_031754288.1
*sod*	F-GACGTGACAACACAGGTTGC R-TACAGCCACCGTAACAGCAG	XM_003449940.5
*il-10*	F-CTGCTAGATCAGTCCGTCGAA R-GCAGAACCGTGTCCAGGTAA	XM_013269189.3
*il-8*	F-GCACTGCCGCTGCATTAAG R-GCAGTGGGAGTTGGGAAGAA	XM_031747075.1
*il-β*	F-TGCTGAGCACAGAATTCCAG R-GCTGTGGAGAAGAACCAAGC	XM_019365841.2
*tnf-α*	F-GAGGTCGGCGTGCCAAGA R-TGGTTTCCGTCCACAGCGT	NM_001279533.1
*mtor*	F-TGCGGAGTATGTGGAGTT R-CATCTCTTTGGTCTCTCTCTGG	XM_019108641.1
*bcln-1*	F-TCTGTTTGATATCATGTCTGG R-TAATTCTGGCACTCATTTTCT	XM_019068185.1
*lc3-II*	F-GGAACAGCATCCAAGCAAGA R-TCAGAAATGGCGGTGGACA	NM199604.1
*atg12*	F-ACAGTACAGTCACTCGCTCA R-AAAACACTCGAAAAGCACACC	XM_019125508.1
*atg5*	F-ATTGGCGTTTTGTTTGATCTT R-TTTGAGTGCATCCGCCTCTTT	XM_019082404.1

*sod*: superoxide dismutase, *cat*: catalase, *gsh-px*: glutathione peroxidase, *il*: interleukin, *tnf-α:* tumor necrosis factor-*α*, *lc3-II*: microtubule-associated proteins 1A/1B light chain, *bcln-1*: beclin-1, *atg*: autophagy, and *mtor*: mechanistic target of rapamycin.

**Table 3:** Growth performance and profitability variables of Nile tilapia (*O. niloticus*) fed diets fortified with Thy and/or ThQ.

Parameter	Experimental Group				*p* Value	SEM
	Control	G1	G2	G3		
Initial body weight (g/fish)	12.61	12.43	12.80	12.65	0.136	0.06
Final body weight (g/fish)	68.07 ^c^	93.47 ^b^	91.65 ^b^	99.67 ^a^	<0.02	15.63
Final weight gain (g/fish)	55.45 ^c^	81.03 ^b^	78.85 ^b^	87.01 ^a^	<0.03	11.20
Total feed intake (g/fish)	81.83	96.80	92.17	82.80	0.09	14.60
Feed conversion ratio	1.48 ^a^	1.19 ^b^	1.17 ^b^	0.95 ^c^	<0.02	0.003
Specific growth rate (%)	2.01 ^c^	2.40 ^ab^	2.34 ^b^	2.46 ^a^	<0.001	0.03
Protein efficiency ratio	2.12 ^c^	2.62 ^b^	2.67 ^b^	3.28 ^a^	<0.001	0.019
Survival (%)	92 ^c^	94 ^b^	95 ^b^	97 ^a^	0.02	4.65
Net profit	0.08 ^c^	0.14 ^ab^	0.13 ^b^	0.14 ^a^	0.04	0.01
Economic efficiency	1.21 ^b^	1.58 ^a^	1.45 ^a^	1.45 ^a^	<0.001	0.16
Feed cost/kg gain	1.18 ^a^	1.05 ^b^	1.11 ^ab^	1.12 ^ab^	0.04	1.33

Mean values with different letters in the same row differ significantly at *p* < 0.05. SEM: standard error of the mean. Fixed costs = 0.06 $, price of one kg diet of G1, G2 and G3 groups; 0.8, 0.95–1.03 and 1.18 $, respectively. Control: tilapia fed basal diets without any supplementations, G1: tilapia fed basal diets with thymol (Thy) supplementation at a concentration of 200 mg/kg diet, G2: tilapia fed basal diets with thymoquinone (ThQ) supplementation at a concentration of 200 mg/kg, diet, and G3: tilapia fed basal diets with Thy/ThQ supplementation at a concentration of 200 mg/kg diet each.

**Table 4:** Hematological, biochemical and immunological markers of Nile tilapia (*O. niloticus*) fed diets fortified with Thy and/or ThQ for 12 weeks.

Parameter	Experimental Group				*p*-Value	SEM
	Control	G1	G2	G3		
Ht (%)	28.88	28.38	29.10	28.70	0.49	0.09
Hb (g/dL)	8.80	9.27	10.01	10.17	0.345	0.34
RBCs (×10^6^/μL)	1.93	2.20	2.10	2.70	0.06	0.08
ALT (U/L)	64.97	62.96	65.80	63.80	0.86	3.25
AST(U/L)	21.27	21.53	21.00	21.16	0.09	1.96
Creatinine (mg/dL)	0.39	0.37	0.38	0.39	0.93	0.002
Urea (mg/dL)	6.33	6.03	6.16	6.03	0.069	0.06
Cholesterol (mg/dL)	77.87 ^a^	79.03 ^a^	72.13 ^b^	67.20 ^c^	0.02	15.30
Triacylglycerol (mg/dL)	52.60 ^a^	53.40 ^a^	47.06 ^ab^	42.70 ^b^	0.03	10.25
Serum lysozyme (μg/mL)	1.03 ^d^	1.32 ^c^	1.57 ^b^	1.98 ^a^	<0.001	0.01
Serum alternative complementary (u/mL)	259 ^c^	271 ^b^	272 ^b^	283 ^a^	0.03	19.36
MPO (μmoL/L, OD 450 nm)	0.51 ^c^	0.50 ^c^	0.75 ^b^	0.90 ^a^	<0.001	0.003
IgM (μg/mL)	29.49 ^d^	30.20 ^c^	35.10 ^b^	37.90 ^a^	<0.001	2.02

Ht: hematocrit, Hb: hemoglobin, RBCs: red blood cells, AST: aspartate transaminase, ALT: alanine transaminase, MPO: myeloperoxidase, and IgM: immunoglobulin M. Mean values with different letters in the same row differ significantly at *p* < 0.05. SEM: standard error of the mean. Control: tilapia fed basal diets without any supplementations, G1: tilapia fed basal diets with thymol (Thy) supplementation at a concentration of 200 mg/kg diet, G2: tilapia fed basal diets with thymoquinone (ThQ) supplementation at a concentration of 200 mg/kg diet, and G3: tilapia fed basal diets with Thy/ThQ supplementation at a concentration of 200 mg/kg diet each.

## Data Availability

The data presented in this study are available upon request from the corresponding author.
